# Prevalence and Grade of Patellar Luxation in Cavalier King Charles Spaniels Attending Primary‐Care Veterinary Practices in Australia

**DOI:** 10.1002/vms3.71026

**Published:** 2026-06-15

**Authors:** Yaeji Son, Marianne D. Keller, Peter Williamson, Rosanne M. Taylor

**Affiliations:** ^1^ Sydney School of Veterinary Science, Faculty of Science University of Sydney Camperdown New South Wales Australia

**Keywords:** Cavalier King Charles Spaniel, dog, musculoskeletal disorder, patellar luxation, prevalence, VetCompass

## Abstract

**Objectives:**

To identify risk factors associated with the prevalence and grade of patellar luxation in Cavalier King Charles Spaniels (CKCSs) attending Australian veterinary practices.

**Methods:**

Patient records (*n* = 321,517) were obtained from VetCompass Australia, data were cleaned, and diagnoses of patellar luxation were identified by keyword search. Two risk factor analyses were performed for dogs over 18 months of age: comparing case and control groups and assessing risk factors influencing the grade of patellar luxation within the case group.

**Results:**

The 10‐year prevalence of patellar luxation in this study was 12.5% (95% confidence interval [CI], 0.12–0.13). The greatest risk of patellar luxation diagnosis was in neutered dogs (odds ratio (OR): 3.00; 95% CI, 1.97–4.71) and dogs weighing 11 to < 13 kg (OR: 2.52; 95% CI, 1.49–4.39). Dogs 10 years or older had the lowest risk (OR: 0.44; 95% CI, 0.27–0.69) of patellar luxation of all age groups. Ruby‐coloured dogs were most likely to be diagnosed with patellar luxation (OR: 2.04; 95% CI, 1.37–3.04). Dogs in Victoria had the highest prevalence of high grade patellar luxation (OR: 1.68; 95% CI, 1.11–2.53) compared to other states. Dogs with bilateral patellar luxation were more likely to be diagnosed with higher grade patellar luxation compared to those with unilateral patellar luxation.

**Clinical Significance:**

Patellar luxation is a prevalent disorder in CKCS and awareness of this condition is important for early examination of at‐risk animals and to avoid breeding from the affected population.

## Graphical abstract

Patella luxation was prevalent in Cavalier King Charles Spaniels attending Australian veterinary practices. Dogs which were younger, neutered, above the breed standard weight, and with ruby‐coloured coats were at increased risk, while dogs with bilateral luxation were more likely to be diagnosed with higher grade luxation

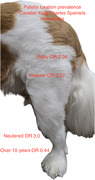

## 1 Introduction

Patellar luxation is a common orthopaedic disorder in dogs whereby the patella is displaced from the trochlear groove causing lameness, skipping, and stiff gait (Harasen 2006). There are four grades of patellar luxation with higher grades indicating increased severity (Campbell et al. 2010). Whilst patellar luxation is unlikely to cause mortality, higher grade luxation is associated with pain, chronic lameness, disability and other orthopaedic disorders associated with joint instability such as cranial cruciate ligament rupture (CCLR) and osteoarthritis (Anderson et al. 2020; Kim et al. 2024). Despite the morbidity of chronic lameness and pain, Grade 1 or 2 patellar luxation are often not reported to veterinarians due to the intermittent nature of the clinical signs (O'Neill et al. 2016; Bosio et al. 2017).

Patellar luxations are likely to have a genetic basis due to abnormal conformation leading to developmental malalignment of the quadriceps, patella, patellar ligament and tibial tuberosity (Nilsson et al. 2018). Previous studies of disease prevalence in the United Kingdom (UK) and Italy have demonstrated small breeds, including the Cavalier King Charles Spaniel (CKCS) breed, as being overrepresented for patellar luxation (Summers et al. 2015; O'Neill et al. 2016; Bosio et al. 2017; Perry and Déjardin 2021). In England, the reported prevalence of patellar luxation in CKCS was 3.8% (95% CI, 2.8%–4.8%; O'Neill et al. 2016). Breed‐specific studies have explored patellar luxation and associated risk factors in Toy Poodles and brachycephalic dog breeds such as Pomeranians, Pugs and French Bulldogs (Wangdee et al. 2017; Maeda et al. 2019; Matchwick et al. 2021). However, while patellar luxation was one of the most reported musculoskeletal disorders in CKCS in the United Kingdom, risk factors associated with patellar luxation, specifically in CKCS, have not yet been explored in detail as it has with other breeds (Summers et al. 2015).

The objectives of this study were to estimate the 10‐year prevalence of patellar luxation and compare demographic information in CKCS attending primary‐care veterinary practices in Australia. The dogs in this study were representative of those attending veterinary practices, that is, both purebred and dogs phenotypically identified as CKCS by a veterinarian. In addition, the study aimed to determine whether some demographic risk factors including sex, neuter status, bodyweight, age, coat colour and location, had an association with higher grade patellar luxation. These findings will provide breeders, owners, and veterinarians with evidence to enhance understanding of patellar luxation in CKCS.

## 2 Materials and Methods

### 2.1 Study Design

The VetCompass Australia (VCA) programme provided the researchers with access to the electronic patient records (EPRs) from Australian primary‐care veterinary clinics (McGreevy et al. 2017) for the years 2008–2017. The raw data recorded during consultations included demographic information about the patient (breed, date of birth, sex, neuter status, age, bodyweight and coat colour) and free‐form clinical notes completed by practitioners that contained health assessment results, summary diagnosis and treatments.

**FIGURE 1:**
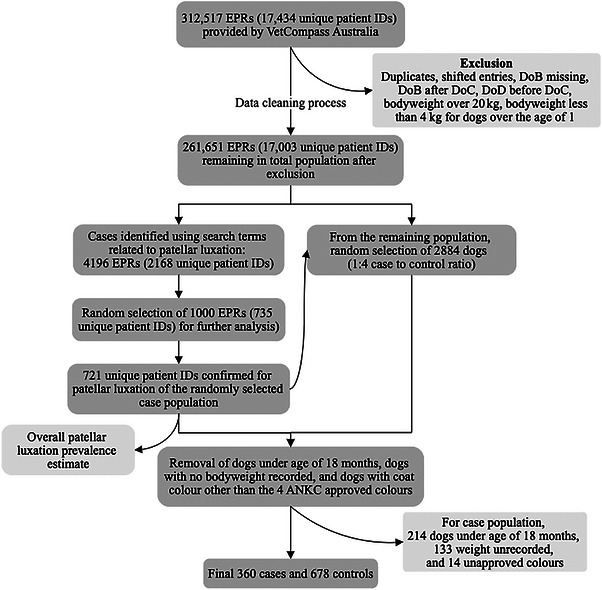
Flow diagram of data exclusion and case and control selection process for CKCS attending primary‐care veterinary clinics in Australia between 2008 and 2017. DoB, date of birth; DoC, date of consultation; DoD, date of death; EPRs, electronic patient records (); ID, identification.

The dataset on CKCSs provided for this VCA‐approved student project on the health conditions in this breed included 321,517 EPRs from 17,434 individual dogs between 1st January 2008 and 31st December 2017. This dataset was evaluated through Microsoft Excel 365 to remove inconsistencies and errors (Figure 1).

### 2.2 Inclusion Criteria

For the selection of cases with potential patellar luxation (medial patella luxation [MPL], lateral patella luxation [LPL]), EPRs that included key search terms, including those for alterative abbreviations used by veterinarians (medial luxating patella [MLP], lateral luxating patella [LLP]; patella*, pat* lux*, slipping pat*, trochlea*, stifle luxation, MPL, MLP, LPL, LLP) were extracted using the filter function on Excel. From the extracted patellar luxation EPRs, a randomly selected sample of 1000 EPRs, sufficient for analysis of the risks associated with demographic factors assuming the expected prevalence of 3.3%, with 1.5% precision and 95% confidence, were extracted using the RAND formula on Microsoft Excel (Figure 1).

The extracted EPRs were manually reviewed to confirm that patellar luxation had been diagnosed. From the confirmed EPRs, the first‐dated diagnosis for each dog was used for further analysis of the case population. Multiple consultations of individual dogs were not included in the analysis to avoid calculations of multiple measurements belonging to the same individual. The 10‐year prevalence of patellar luxation was estimated from the case population and total population accounting for subsampling by using the proportion of confirmed cases within a randomly selected sample and applying it to the total case population. VeNom codes, specialised nomenclature for veterinary conditions, were retrospectively assigned to the case entries related to the stifle, diagnosed during the first‐dated consultation and as described in Venom Coding Group (2024). Further information was extracted from clinical notes such as the stifle affected (right, left, both, or unspecified), type of patellar luxation (medial, lateral or unspecified), grade of patellar luxation (I–IV/IV, or unspecified grade) and treatment. It was assumed that the grade reported by the veterinarian was based on the OFA standard however no measures of inter‐observer variability were available for analysis. If no grade was reported ‘unspecified’ grade was recorded. If the condition was bilateral, the diagnosis was coded as bilateral and assigned to the leg with the higher grade of the two stifles. Cases were allocated to one of five categories: unilateral right, unilateral left, bilateral right, bilateral left, and unspecified.

From the remaining dogs that were not identified as potential patellar luxation cases, 2884 dogs were randomly selected to give a 1:4 ratio of cases to controls for initial inclusion in the risk factor analysis (Figure 1). Dogs under the age of 18 months were removed from both the case and the control population (Figure 1). In addition, dogs with no bodyweight recorded and dogs with coat colours other than the Dogs Australia recognised breed colours (Blenheim, black and tan, ruby, and tricolour) were removed from the population (Figure 1). With these removals, there were 360 cases and 678 controls remaining, an approximate 1:2 ratio of case to control (Figure 1). The final control group was re‐examined to ensure that their clinical notes did not contain the previously outlined search terms relating to patellar luxation. The 360 CKCS from the case group were further examined for risk factors associated with the grade of patellar luxation.

### 2.3 Outcome and Explanatory Variables

Two outcome variables used were the presence and overall grade of patellar luxation. For the first risk factor analysis between patella luxation case and control (Tables 1 and 2), six explanatory variables were used including *sex*, *neuter status*, *bodyweight*, *age*, *coat colour* and *location. Bodyweight* (kg) was categorised into five groups (0 to < 7, 7 to < 9, 9 to < 11, 11 to < 13 and >= 13) and *age* (years) into four groups (1.5–3, 4–6, 7–9 and >= 10). Body condition scores were not consistently available, so bodyweight was analysed independently.

**TABLE 1:** Descriptive and univariate logistic regression results for CKCS with (case) and without (control) patellar luxation in dogs, over 18 months of age, attending primary‐care veterinary clinics in Australia between 2008 and 2017.

Variable	Category	Case no. (%)	Control no. (%)	Odds ratio	95% CI	*p* value
Sex	Male	175 (48.61)	332 (48.97)	Base		0.91
	Female	185 (51.39)	346 (51.03)	1.01	0.79–1.31	
Neuter status	Entire	28 (7.78)	140 (20.65)	Base		< 0.001^*^
	Neutered	332 (92.22)	538 (79.35)	3.09	2.04–4.82	
Bodyweight (kg)	0 to < 7	25 (6.94)	92 (13.57)	Base^a^		< 0.01^*^
	7 to < 9	74 (20.56)	161 (23.75)	1.69	1.02–2.89	
	9 to < 11	103 (28.61)	192 (28.32)	1.97	1.21–3.32	
	11 to < 13	82 (22.78)	118 (17.40)	2.56^b^	1.53–4.38	
	>= 13	76 (21.11)	115 (16.96)	2.43	1.45–4.18	
Age (years)	1.5–3	124 (34.45)	185 (27.29)	Base^b^		< 0.01*
	4–6	133 (36.94)	234 (34.51)	0.85	0.62–1.16	
	7–9	70 (19.44)	145 (21.39)	0.72	0.49–1.04	
	>= 10	33 (9.17)	114 (16.81)	0.43^a^	0.27–0.67	
Coat colour	Blenheim	174 (48.33)	400 (59.00)	Base^a^		< 0.001*
	Black and tan	35 (9.72)	71 (10.47)	1.13	0.72–1.75	
	Ruby	63 (17.50)	68 (10.03)	2.13^b^	1.44–3.14	
	Tricolour	88 (24.45)	139 (20.50)	1.46	1.05–2.00	
Location	NSW	107 (29.72)	216 (31.86)	Base^a^		0.72
	QLD	151 (41.95)	269 (39.67)	1.13^b^	0.84–1.54	
	VIC	102 (28.22)	193 (28.47)	1.07	0.76–1.49	

^a^Lowest OR for patellar luxation compared to other categories of the same variable.

^b^Highest OR for patellar luxation.

* Significant findings (*p* < 0.05).

**TABLE 2:** Final multivariate binary logistic regression model comparing risk factors for case and control groups in CKCS, over 18 months of age, attending primary‐care veterinary clinics in Australia between 2008 and 2017.

Variable	Category	Case no. (%)	Control no. (%)	Odds ratio	95% CI	*p* value
Neuter status	Entire	28 (7.78)	140 (20.65)	Base		< 0.001
	Neutered	332 (92.22)	538 (79.35)	3.00	1.97–4.71	
Bodyweight (kg)	0 to < 7	25 (6.94)	92 (13.57)	Base^a^		< 0.01
	7 to < 9	74 (20.56)	161 (23.75)	1.60	0.95–2.76	
	9 to < 11	103 (28.61)	192 (28.32)	1.96	1.19–3.33	
	11 to < 13	82 (22.78)	118 (17.40)	2.52^b^	1.49–4.39	
	>= 13	76 (21.11)	115 (16.96)	2.34	1.38–4.09	
Age (years)	1.5–3	124 (34.45)	185 (27.29)	Base^b^		< 0.01
	4–6	133 (36.94)	234 (34.51)	0.83	0.60–1.15	
	7–9	70 (19.44)	145 (21.39)	0.68	0.47–1.00	
	>= 10	33 (9.17)	114 (16.81)	0.44^a^	0.27–0.69	
Coat colour	Blenheim	174 (48.33)	400 (59.00)	Base^a^		< 0.01
	Black and tan	35 (9.72)	71 (10.47)	1.29	0.81–2.03	
	Ruby	63 (17.50)	68 (10.03)	2.04^b^	1.37–3.04	
	Tricolour	88 (24.45)	139 (20.50)	1.51	1.08–2.11	

^a^ Lowest OR for patellar luxation compared to other categories of the same variable.

^b^Highest OR for patellar luxation.

The second risk factor analysis explored the grade of patellar luxation (Tables 3 and 4), which was tested against seven explanatory variables with *stifle(s) affected* added onto the previous variables. The categories for *age* were adjusted from the first risk factor analysis into four different groups (1.5–2, 3–5, 6–8 and >= 9) after the assessment of the contingency table that confirmed this category as a suitable proportion for the ordinal outcome.

**TABLE 3:** Univariate ordinal logistic regression analysis of the grade of patellar luxation against demographic risk factors in CKCS, over 18 months of age, attending primary‐care veterinary clinics in Australia between 2008 and 2017.

Variable	Category	Estimate	Standard error (SE)	Odds ratio	95% CI	*p* value
Sex	Male	0	0	Base		0.05
	Female	0.34	0.17	1.40	1.00–1.98	
Neuter status	Entire	0	0	Base		0.40
	Neutered	0.23	0.28	1.26	0.74–2.18	
Bodyweight (kg)	0 to < 7	0	0	Base		0.22
	7 to < 9	0.33	0.35	1.39	0.70–2.75	
	9 to < 11	0.46	0.34	1.59	0.81–3.11	
	11 to < 13	0.48	0.35	1.62^b^	0.81–3.26	
	>= 13	−0.15	0.38	0.86^a^	0.41–1.81	
Age (years)	1.5–2	0	0	Base		0.29
	3–5	−0.15	0.21	0.86	0.57–1.30	
	6–8	−0.45	0.24	0.64^a^	0.40–1.02	
	>= 9	0.008	0.31	1.01^b^	0.55–1.84	
Coat colour	Blenheim	0	0	Base^a^		0.27
	Black and tan	0.53	0.28	1.70^b^	0.97–2.97	
	Ruby	0.04	0.24	1.04	0.65–1.66	
	Tricolour	0.21	0.23	1.23	0.79–1.93	
Location	QLD	0	0	Base^a^		0.06
	NSW	0.31	0.21	1.37	0.90–2.07	
	VIC	0.48	0.21	1.62^b^	1.08–2.44	
Stifle(s) affected	Both	0	0	Base^b^		< 0.01^*^
	Right	−0.65	0.21	0.52^a^	0.35–0.79	
	Left	−0.55	0.21	0.58	0.38–0.87	

^a^Lowest OR for high grade patellar luxation compared to other categories of the same variable.

^b^Highest OR for high grade patellar luxation.

* Significant findings (*p* < 0.05).

**TABLE 4:** Final multivariate ordinal logistic regression analysis of the grade of patellar luxation against demographic risk factors in CKCS, over 18 months of age, attending primary‐care veterinary clinics in Australia between 2008 and 2017.

Variable	Category	Estimate	SE	Odds ratio	95% CI	*p* value
Location	QLD	0	0	Base^a^		< 0.05
	NSW	0.31	0.21	1.37	0.90–2.07	
	VIC	0.52	0.21	1.68^b^	1.11–2.53	
Stifle(s) affected	Both	0	0	Base^b^		< 0.01
	Right	−0.66	0.21	0.52^a^	0.34–0.78	
	Left	−0.58	0.21	0.56	0.37–0.85	

^a^ Lowest OR for high grade patellar luxation compared to other categories of the same variable.

^b^ Highest OR for high grade patellar luxation.

### 2.4 Statistical Analysis

All data analyses were performed on R Studio version 1.3.1093. Contingency tables were produced using ‘tabyl’ on the janitor package for all explanatory variables against both binary and ordinal outcomes to determine the acceptable proportion of categories.

For the first risk factor analysis comparing case and control, a binary univariate logistic regression model was produced using the generalised linear model with significance set at *p* < 0.05. From the univariate model, risk factors with liberal associations (*p* < 0.2) were included for multivariate modelling. A manual forward stepwise method was used to produce the final multivariate binary logistic regression model. During this process, any variables that were not significant (*p* > 0.05) were taken out of the model. The Hosmer–Lemeshow test was performed using the ‘performance_hosmer’ function to assess the goodness‐of‐fit of the model.

The second risk factor analysis, assessing risk factors relating to the grade of patellar luxation, was explored with ordinal logistic regression using the ‘MASS;;polr’ function on the mass package implemented in R (Venables and Ripley 2002). This regression model demonstrated risk factors that are likely to be associated with higher grade patellar luxation. The same method was followed for multivariate modelling and the manual forward stepwise method to produce the final multivariate ordinal logistic regression. For confirmation of the final model, a Brant test implemented in R, was used to test for parallel regression assumption.

In regard to both risk factor analyses, variables that were included in the final model with more than two categories were assessed with ‘emmeans’ for a pairwise comparison.

## 3 Results

### 3.1 Prevalence Estimate

From the total population of CKCS, 4195 EPRs (2167 dogs) were identified by the search terms as likely to have patellar luxation. A random sample of 735 dogs (1000 EPRs) was manually checked to validate cases and 721 dogs (98.1%) were confirmed to be positive for patellar luxation. The estimated overall 10‐year prevalence of patellar luxation was 12.5% (95% CI, 0.12–0.13) with consideration of the effects of subsampling.

More CKCS from those investigated as likely to have patella luxation were diagnosed with medial patellar luxation (*n* = 270, 37.4%) compared to lateral patellar luxation (*n* = 11, 1.5%). The type of patellar luxation for the remaining 440 dogs (61.1%) was unspecified on the clinical notes. The grade of patellar luxation was recorded in 482 dogs (66.9%) and it was assumed that the standardised Orthopaedic Foundation for Animals (OFA) grading was used (OFA 2026). The grade of patellar luxation for the remaining 239 dogs (33.1%) was unspecified.

### 3.2 Case–Control Demographics

The initial univariate risk factor analysis for selected cases of patellar luxation and controls are presented in Table 1. The first univariate logistic regression indicated *neuter status*, *bodyweight, age* and *coat colour* to have a statistically significant effect (*p* < 0.01) on the presence of patellar luxation. The results for *sex* and *location* were not significant (Table 1) and these variables were excluded from this final model.

Table 2 shows the final multivariate binary logistic regression model developed after three rounds of manual stepwise assessment. The variables considered for multivariate analysis from the univariate model were those with significance at *p* < 0.05. Neutered dogs had three times higher odds of having patellar luxation compared to entire dogs (95% CI, 1.97–4.71; *p* < 0.001). In terms of bodyweight, the highest odds of patellar luxation were seen in dogs weighing between 11 and 13 kg (OR: 2.52; 95% CI, 1.49–4.39; *p* < 0.01) and a trend was observed with dogs of lower bodyweight having decreased OR of patellar luxation. The odds of being diagnosed with patellar luxation decreased with age, where dogs more than 10 years of age had significantly lower odds compared to dogs between 18 months and 3 years of age (OR: 0.43; 95% CI, 0.27–0.67; *p* < 0.01). Lastly, ruby‐coloured dogs were at increased risk of patellar luxation (OR: 2.04; 95% CI, 1.37–3.04; *p* < 0.01) compared to dogs of other coat colours. Tricoloured dogs had 1.51 times higher odds of patellar luxation compared to Blenheim dogs (95% CI, 1.08–2.11; *p* < 0.01). The Hosmer and Lemeshow goodness‐of‐fit test for the final model produced a *p* value of 0.28, indicating a good fit.

### 3.3 Grade of Patellar Luxation

The second model assessing the grade of patellar luxation demonstrated two variables, *stifle(s) affected* and *location*, as having significant association with higher grade patellar luxation (Tables 3 and 4).

Table 3 shows the univariate ordinal logistic regression assessing risk factors associated with the grade of patellar luxation in CKCS. From the risk factors, only three variables with liberal associations (*p* < 0.2), *sex*, *location* and *stifle(s) affected*, were considered for the multivariate model. In the univariate model, female CKCS had 1.4 times higher odds (95% CI, 1.00–1.98; *p* = 0.05) of being diagnosed with a higher grade of patellar luxation compared to males. This finding, whilst considered for the multivariate model, was not included in the final multivariate logistic regression as the association was not strong enough.

The final multivariate logistic regression for this model, investigating the grade of luxation (Table 4), included *location* (*p* = 0.04) and *stifle(s) affected* (*p* = 0.001) under the criteria of *p* < 0.05. The final model showed good fit with proportional odds of assumption being held true on the Brant test (omnibus *p* = 0.17).

There was a higher grade patellar luxation diagnosed in the population living in Victoria (VIC; OR: 1.68; 95% CI, 1.11–2.53; *p* < 0.05) compared to Queensland (QLD) and New South Wales (NSW; OR: 1.37; 95% CI, 0.90–2.07; *p* < 0.05). Pairwise comparison between the three states showed significant differences between VIC and QLD (*p* = 0.0367). Additionally, the odds for a severe patellar luxation were lower in CKCS with right stifle affected (OR: 0.52; 95% CI, 0.34–0.78; *p* < 0.01) or left stifle affected (OR: 0.56; 95% CI, 0.37–0.85; *p* < 0.01) compared to those with both stifles affected. Since a pairwise comparison within the three levels indicated no significant difference between the right or the left stifle, it can be concluded that CKCS with bilateral patellar luxation had higher odds of being diagnosed with higher grade patellar luxation compared to those with unilateral patellar luxation.

## 4 Discussion

This is the first study to identify risk factors associated with patellar luxation in CKCS and reports high prevalence of this condition in Australia. There was a high prevalence rate (12.5%) of patellar luxation diagnosed in CKCS attending primary‐care veterinary clinics in Australia between 2008 and 2017. Our result was higher compared to previous studies which recorded a prevalence rate between 3.3% and 3.8%, in CKCS living in the United Kingdom, using similar methods analysing EPRs (Summers et al. 2015; O'Neill et al. 2016). The UK multi‐breed study by O'Neill et al. (2016) had a lower case confirmation rate of patellar luxation (42.7%) than the rate of case confirmation in the current study (98.1%), despite similar search terms and methodology. The observed difference may reflect variation in clinical recording between United Kingdom and Australia primary‐care practices whereby the former reports animals without patellar luxation as ‘no patellar luxation’ compared to the latter that typically records this information only when a luxation is present. Consequently, database searches using terms such as ‘patellar luxation’ may retrieve more false positives in UK records, where cases explicitly noted as having no luxation are still captured, resulting in a lower confirmation rate for true patellar luxation.

The current study reported a higher frequency of CKCS diagnosed with medial patellar luxation (37.4%) compared to lateral patellar luxation (1.5%), consistent with the high prevalence of medial patellar luxation reported in other small dog breeds (Roush 1993).

### 4.1 Risks Related to Prevalence

Several risk factors for patellar luxation were identified in this population. The results indicate that neutered dogs had three times higher odds of patellar luxation compared to entire dogs. This result was consistent with studies in all dog breeds which reported neutered dogs having 2.1–3.1 times higher odds (O'Neill et al. 2016; Vidoni et al. 2006). Neutering may influence orthopaedic disorders through the removal of gonadal hormones disrupting the closure of growth plates, thus leaving the joints vulnerable (Hart et al. 2016). Furthermore, recent study has shown that there is an increased risk of other joint disorders such as CCLR with early neutering (before 12 months of age; DeForge et al. 2024). However, as the current study did not examine the age of neutering, the temporal relationship between neutering and the onset of patellar luxation cannot be determined. Another consideration is that neuter status has been associated with higher body condition score in many breeds, with studies reporting neutered dogs to have an increased risk of obesity (McGreevy et al. 2005). Obesity contributes to the progression of joint disorders such as osteoarthritis through both mechanical factors, such as heavier load on weight‐bearing joints, and metabolic factors, including expansion of adipokines like leptin (Anderson et al. 2020; Dumond et al. 2003). It is acknowledged that neutered dogs may appear overrepresented because their owners more frequently seek veterinary care, increasing the likelihood of diagnosis, whereas patellar luxation may remain undetected in entire dogs presenting less often; this may reflect confounding by indication.

High bodyweight does not necessarily suggest a high body condition score as the height and bone mass differs in individual dogs of this breed. Measurement of bodyweight and body condition score should be part of future primary‐care visits. Although the dogs in this study were recognised as CKCS, they were drawn from the pet population where dogs that are pedigree, crossbred, or not bred to meet breed standards are likely to be present. Since the body condition score was infrequently recorded, bodyweight was compared to the Australian breed standards. Dogs Australia indicates a CKCS weight range between 5.4 and 8.2 kg as desirable (Dogs Australia 2008). The current study showed that dogs over the breed‐relative bodyweight (over 8.2 kg) were more likely to be diagnosed with patellar luxation compared to those under the recommended bodyweight. The increased pressure put on the stifle in dogs with bodyweight higher than breed‐relative weight may be a major contributor to the development of patellar luxation. However, this result is in opposition to the findings of the UK study, which reported dogs below the breed‐relative bodyweight to have a higher risk of patellar luxation, potentially due to the reduced muscle mass in lighter dogs (O'Neill et al. 2016).

Age had an association with the prevalence of patellar luxation. Patellar luxation and similar lifelong disorders, often first recognised in young dogs, compromise welfare through chronic pain and progressive joint damage, predisposing to CCLR and osteoarthritis (O'Neill et al. 2016). Dogs under the age of 18 months of age were excluded from the study to remove potential skewing that could occur. Skeletally immature dogs may exhibit certain levels of non‐pathologic joint instability which could have given false positive results (Vasseur 1993). Older dogs were at lower risk (OR: 0.44–0.83) of having their first diagnosis of patellar luxation compared to younger dogs over 18 months of age, which was in agreement with previous multi‐breed studies (O'Neill et al. 2016; Alam et al. 2007). In addition, 214 of 721 dogs confirmed with patellar luxation were under the age of 18 months, although they were excluded from the study as they had not reached mature body size (Figure 1). Younger dogs are more likely to be diagnosed with patellar luxation than older dogs as this condition is a developmental disease with a heritable basis (Roush 1993). A recent study by Nagahiro et al. (2023) concluded that the quadriceps muscle length (QML) to femoral length (FL) ratio (QML/FL) may have an impact on medial patellar luxation in small breed dogs; with younger dogs having a shorter QML.

This study is the first to report on an association between coat colour and the prevalence of patellar luxation. Ruby‐coloured CKCS had the highest odds (OR: 2.04; 95% CI, 1.37–3.04; *p* < 0.01) of patellar luxation compared to other colours. This significant finding has a robust effect size, reducing the likelihood of Type II error. Research on the impact of coat colour on musculoskeletal conditions, including patellar luxation, is lacking. The current study did not explore any genetic or molecular evidence behind this correlation, however some speculations can be made based on previous studies that examined association between coat colour and musculoskeletal diseases. Ruby CKCS have a homozygous recessive genotype for the extension (E) locus, determining black (E/E or E/e) or red (e/e) pigmentation, and homozygous dominant or heterozygous for the S locus for the solid (S/S or S/s) or parti (s/s) colouration (Everts et al. 2000). A DNA variant of the melanocortin 1 receptor (MC1R) gene (e) that promotes the production of pheomelanin, responsible for red pigmentation, has been widely expressed in musculoskeletal system especially in muscle fibres surrounding the femur and patella (Thomas et al. 2018). MC1R gene has also been reported to overlap with regions of genome that are biologically significant to CCLR risks in Labrador Retrievers (Baker et al. 2021). The Labrador Retriever findings suggested that this relationship may provide an explanation as to yellow Labrador Retrievers, which lack MC1 receptors, being predisposed to inflammation and CCLR (Baker et al. 2021). Ruby CKCS also lack MC1 receptors, potentially helping to explain their increased risk for patellar luxation. However, this study did not consider whether patients had concomitant conditions such as CCLR which could have been a confounder in this interpretation. Furthermore, tricoloured CKCS that have a genotype [(E/E or E/e) and s/s], opposite to ruby dogs, had the second‐highest odds (OR: 1.51) of patellar luxation, whilst Blenheim dogs, that also carry pheomelanin and white, were underrepresented for the prevalence of patellar luxation. Further genetic studies are needed to explore the relationship between coat colour and patellar luxation in CKCS.

### 4.2 Risk Related to Grade

In assessing risk factors that increase the population risk for higher grade of patellar luxation, two variables, *location* and *stifle(s) affected*, were included in the final model. Whilst the effect of location was not significant regarding the prevalence of patellar luxation, the location was associated with the higher grade patellar luxation within the case population. CKCS living in Queensland had the lowest risk of being diagnosed with high‐grade patellar luxation, whereas CKCS in VIC had the highest risk with an odds ratio of 1.68 compared to dogs in QLD. It may be speculated that localised genetic differences may arise from the popular sire effect (Haynes 1915). Furthermore, different levels of awareness and action to reduce patellar luxation in CKCS between different kennel clubs may have had an influence. The CKCS Club of QLD highlights patellar luxation as a major health risk for CKCS on their website and encourages certification by the orthopaedic foundation of animals (OFA; CKCS Club of Qld 2017). This information was absent in the website information provided by the CKCS Club of NSW and was only recently added in the CKCS club of VIC, which may indicate a lower or slower awareness of this issue in NSW and VIC compared to QLD. There is also a possibility that the higher odds of higher grade patellar luxation observed in VIC may partly reflect regional differences in diagnostic practices or case recording.

In addition to the location, the type of patellar luxation, whether bilateral or unilateral, had a significant effect on the grade. Dogs with bilateral patellar luxation were more likely to be diagnosed with a higher grade patellar luxation compared to those with unilateral patellar luxation. Dogs with higher grade patella luxation may have more severe underlying congenital or developmental conformational abnormalities, which could explain the results obtained from the current study. Exclusion criteria for cases and controls did not include other stifle or orthopaedic conditions which may have affected patella function.

### 4.3 Limitations

The limitations of this study include the variation in the quality of VetCompass data with incomplete or lacking information in clinical notes completed by the large number of participating practices. The limited detail in clinical notes posed challenges for consistent data coding and monitoring for variation in the grade assigned by veterinarians. Additionally, there is a possibility that the EPRs of dogs in the control population may have had inaccuracies, for example, unreported patella luxation resulting in misclassification bias. Greater peer scrutiny of clinical notes to ensure consistent entries for all body systems would assist in reducing the challenge in coding diagnosed conditions and improve the accuracy of the results. Furthermore, consistent recording of the grade of patellar luxation based on the OFA grading system would assist research and clinical monitoring, although inter‐observer variability may still exist (OFA 2026).

More than 200 veterinary practices participated in VCA and among those providing 10 years of EPRs these 48 practices were in the three most populous states.

The dogs involved in this study were not all pedigree CKCS. They may have the phenotypic appearance of a CKCS, but it is possible that the group included some crossbreds and dogs that were not bred to breed standards. For future studies, it is recommended that body condition scores are recorded along with bodyweight for the determination of overweight condition and its impact on patellar luxation.

The current study focused exclusively on cases of patellar luxation and did not exclude concomitant musculoskeletal diseases such as CCLR and osteoarthritis from the control group. The heterogeneity in the control group may have consequently increased variability and misclassification bias, potentially obscuring associations specific to patellar luxation.

### 4.4 Future Directions and Implications

The results of this study exploring the prevalence and grade of patellar luxation in CKCS open areas for further research such as the genetic relationship between coat colour and patellar luxation. As mentioned previously, confounders such as CCLR should be excluded from future studies to reduce variability. Moreover, exploring longitudinal data that follows individual dogs over time would also be an area for future research whereby orthopaedic disorders that can occur as sequelae to patellar luxation such as CCLR and osteoarthritis can be analysed with risk factors.

Our findings confirm the importance of early detection of patellar luxation in young CKCS and increased awareness of the higher risk of this condition in neutered, overweight, and ruby‐coloured dogs. Recent research has shown growing interest in the relationship between the age of neutering and the development of CCLR (DeForge et al. 2024). The present study suggests that patellar luxation may also be influenced by neutering. Further studies exploring the impact of neutering age on the development of patellar luxation would be valuable to determine whether a similar association exists as seen with CCLR. Weight management programs are needed for overweight dogs to reduce the risk of patellar luxation and associated joint disorders, such as cartilage erosion and osteoarthritis. MC1R agonist therapy has been shown to protect joints and cartilage through induction of synovial fibroblast senescence (Montero‐Melendez et al. 2020). Studies to assess the feasibility and effectiveness of this therapy in dogs may contribute to control and prevention of arthritic changes associated with patellar luxation in CKCS. As ruby CKCS have mutations in MCR1 associated with impaired melanin production, it is postulated that they may also have diminished synovial fibroblast senescence in response to MC1R agonists.

The strong breed association indicates that genetic factors are involved, allowing the opportunity to select away from this condition. Consequently, the Canine Inherited Disorders Database advised breeders to not breed dogs that have been affected by patellar luxation (Canine Inherited Disorders Database 2011). Some clubs enforce this advice as an official rule for breeding such as the American CKCS Club (American CKCS Club 2024), however, many other clubs either do not list patellar luxation as one of the major health problems in this breed, such as the CKCS Club of NSW and UK CKCS Club. To reduce the risk of patellar luxation in CKCS living in Australia, revision of the breeding standards for stifle conformation is necessary along with breeding restrictions that require OFA certification of normal patella function.

## 5 Conclusions

The findings of this study indicated patellar luxation as a highly prevalent disorder present in CKCS living in Australia with a rate of 12.5%. The current study did not find a statistically significant difference between sex, unlike many studies that have reported an over‐representation of female dogs with patellar luxation over males. Being young, neutered, overweight, or ruby‐coloured increased the risk of diagnosis of patellar luxation in CKCS, while the higher grade patellar luxation was associated with two factors, location and stifle(s) affected. Dogs living in Victoria and dogs with bilateral patellar luxation were at the highest risk of higher grade patellar luxation. Active breed control strategies through early testing for patellar luxation and avoiding breeding from affected populations are necessary to reduce the overall prevalence of patellar luxation in dogs, especially in predisposed breeds such as CKCS.

## Author Contributions

**Rosanne M. Taylor and Yaeji Son**: conceptualisation; **Rosanne M. Taylor and Yaeji Son**: methodology; **Yaeji Son**: data curation; **Yaeji Son**: writing – original draft preparation; **Yaeji Son, Rosanne M. Taylor and Marianne D. Keller**: writing – review and editing; **Rosanne M. Taylor, Marianne D. Keller and Peter Williamson**: supervision. All the authors have read and agreed to the published version of the manuscript.

## Funding

Funding for the VetCompass project was provided by the seven veterinary schools of Australia; Charles Sturt University, James Cook University, Murdoch University, University of Adelaide, University of Melbourne, University of Queensland and University of Sydney. The Australian government provided additional funding for VetCompass project managers. No funding was provided for the current study.

## Ethics Statement

This study was approved by the University of Sydney Human Ethics Committee, New South Wales, Australia (Project title: VetCompass Australia; project number 2013/919).

## Conflicts of Interest

The authors declare no conflicts of interest.

## Data Availability

Data supplied by VetCompass Australia, to whom requests should be directed.
